# Is a Food Shortage Coming to the Western Balkans?

**DOI:** 10.3390/foods11223672

**Published:** 2022-11-16

**Authors:** Tatjana Brankov, Bojan Matkovski

**Affiliations:** Department of Agricultural Economics and Agribusiness, Faculty of Economics in Subotica, University of Novi Sad, 24000 Subotica, Serbia

**Keywords:** Western Balkans, food security, self-sufficiency

## Abstract

In the wake of the international food crisis, many countries are paying more attention to food self-sufficiency to protect themselves from instability in the global food markets. Western Balkan region and the rest of the world are facing an array of challenges, including inflation and rising food prices. Recognizing the importance of producing sufficient food to cover a country’s needs in circumstances of increasing risk of trade disruptions due to war or political tensions, this article aims to find the level of food self-sufficiency in the Western Balkan countries. The self-sufficiency ratio is calculated for different food groups and individual products over a 14-year period (2006–2019) based on the FAO data and calculation method. Also, using panel data, the impact of different factors—arable land, rural population, fertilizers efficiency, temperature change, precipitation, and GDP per capita change—on cereals self-sufficiency is estimated. Results showed that in the covered pre-crisis period, the Western Balkans achieved a satisfactory level of food self-sufficiency, suggesting that the region is ready to respond to future challenges. Four indicators positively and significantly affect cereal self-sufficiency: arable land, rural population, fertilizers efficiency and GDP per capita change, while one indicator—temperature change—had a negative and significant effect. This article can serve as a basis for post-crisis research on the topic.

## 1. Introduction

All five Western Balkan countries (Albania, Bosnia and Herzegovina, North Macedonia, Montenegro, and Serbia) have experienced a very turbulent history. For the former states of Yugoslavia, it included a closed economy, wars, and inflation in the 1990s, breaking up the state of Yugoslavia as well as institutional and economic reforms in the newly created states. Albania also had to implement several reforms, considering it was one of the most closed economies. Although more than two decades have passed since then, the results of the region’s transition are not satisfactory. Its’ progress toward becoming a functioning market economy is modest and uneven. Overall living standards decreased while the unemployment rate increased [1]. Consequently, the region experienced mass emigration—predominantly a phenomenon known as the “brain drain”—emigration of highly educated young people in search of higher-paying jobs or better working conditions [2]. For example, North Macedonia has lost 10% of its population in the last 20 years; Albania roughly 37% of its population in the past three decades [3]; 500,000 youth left Serbia in the period 1991–2001, and 240,000 citizens in the period 2008–2015 [4]. As a whole, the region is in a fragile state due to the interaction of complex internal and external challenges [5].

As with the rest of the economy, the Western Balkans’ agriculture still lags behind the European Union (EU) in terms of productivity [6,7], although the share of utilized agricultural area (UAA) in the Western Balkans in the total area is bigger than in the EU. The region uses almost half, while the EU uses 38.4% of its territory for agriculture [8]. A decline in farmland, aging and decline of the rural population, youth migration from rural areas, land abandonment, and urban sprawl are the key trends in the Western Balkans in recent years [9]. For example, Serbia does not use 424,054 hectares or every ninth hectare of available agricultural land [10]. Some reasons for the dramatic situation in the leading region supplier country are the use of agricultural land for non-agricultural purposes and corruption in the land sector [11]. Various reasons impact its inefficiency and low productivity, including privatization, land reforms, farm restructuring, budget constraints, lack of incentives, and disorganized food supply chains [12]. As a result, the average farm-size are lower, and the agricultural population is bigger, while agriculture, forestry, and fishing contribute considerably more to gross value added (GVA) in Western Balkan than in the EU [8]. Western Balkans’ share of total household expenditure (as a proxy of income) on food is significantly higher than in the EU. Household spending, on average, 42% of the income on food in Albania, 30% in Bosnia and Herzegovina, 44% in North Macedonia, 32% in Montenegro, and 28% in Serbia [13]. In contrast, households in the EU spent 13.0% of their total consumption expenditure on food and non-alcoholic beverages in 2019 [14].

Western Balkan countries have a shared strategic objective—accession to the EU. They are in the various stage of the accession—the most advanced are Montenegro and Serbia, next North Macedonia and Albania, while for Bosnia and Herzegovina, on 12 October 2022, the European Commission recommended that candidate status be granted. However, despite the objective that unites the whole region and the EU, exposed to two types of pressures, often contradictory—the EU’s requirements and domestic interest groups—Western Balkan countries rather choose domestic political economy interests than alignment with EU policy [15].

If turning to one’s own national interests at the expense of the EU was unacceptable in the past years, today, it is more understandable. Hunger is stalking—the world is moving backward on eliminating hunger and malnutrition, away from the intended Sustainable Development Goals (SDGs). Food insecurity and malnutrition due to conflicts, climate extremes, economic shocks, and growing inequalities significantly increased. The number of people affected by hunger globally rose to as many as 828 million in 2021, an increase of 46 million since the COVID-19 pandemic started (2020); 350 million more were moderately or severely food insecure in 2021; a gender gap in food insecurity rose for more than 1% in 2021 compared to 2020 [16].

The conflict in Ukraine deepened the problem—it has blocked ports; the price of freight and fertilizer costs increased due to rising fuel prices; social unrest and political violence are rising, adding to risk for the global economy; the number of internally displaced and refugees increased; many countries have empty reserves (due to spending during the pandemic); the already-climbing world food prices exploded due to de facto export blockade or the temporary bans. The dimensions of the consequences are understandable, knowing that Ukrainian and Russian wheat and barley account for almost a third of global exports, sunflower oil more than 70%, and Russia is the top global fertilizer producer [17]. Moreover, some Western Balkan countries depend on Russia’s and Ukraine’s export of grains, vegetable oils, and fertilizers. At the same time, all of them are exposed to the indirect impact of the global trade disruption in key commodities, including energy [18]. Fertilizers represent the best example of dependence on imports. According to IFASTAT [19] data about the total N + P_2_O_5_ + K_2_O production and imports in 2020, Serbia produces about 30%, and Bosnia and Herzegovina about 15% of its total fertilizers needs. The rest of the region—Montenegro, Albania, and North Macedonia completely rely on imports. Dependence on imports in an open market that functions without interruption is not problematic. But in light of new events and the current crisis, all countries, including the region, dependent on imports will face various problems. The fertilizer supply shock is expected to cause severe consequences in the Western Balkan’s food system. The farmers will either reduce their use, which will result in a decrease in yields or, like American farmers, they will begin to reduce the area under corn and wheat and increase the area under soybeans, which require less fertilizer [20]. In addition, the supply in Western Balkan countries is actually at risk from the global/regional shocks because the EU is the main partner both in exports (81.0%) and imports (57.9%) [21]. As a consequence of the current crisis embodied in the higher cost of food and energy, imports have sharply increased, while the region’s export boom has begun to slow [18].

Due to supply-side pressures, headline inflation in the region started picking up in the second half of 2021. As a result, the headline consumer price index (CPI) growth in August 2022 ranged from 16.8% to 8% across the region (16.8% in North Macedonia, 16.7% in Bosnia and Herzegovina, 15% in Montenegro, 13.2% in Serbia, and 8% in Albania). In all countries, food price inflation contributed the most to headline inflation: the cost of food in August of 2022 increased over the same month in the previous year for 25.5% in Montenegro, 25.1% in North Macedonia, 24.68% in Bosnia and Herzegovina, 20.4% in Serbia, and 14.9% in Albania [22]. Considering the current food crises, Food and Agricultural Organization (FAO) has recommended a substantive shift in global agricultural policies. The countries should redirect domestic support towards sustainable farming and nutritious foods, enhance the diversity of their domestic production bases, boost locally grown food to make agriculture more resilient, increase agriculture efficiency through increasing fertilizer effectiveness, etc. [23].

In this regard, the **main objective** of this paper is to find the Western Balkan region’s level of food self-sufficiency. Following the FAO recommendations, the additional objective of this paper is to analyze the inputs use effectiveness in the Western Balkans. Finally, this paper analyses the factors influencing the level of cereals self-sufficiency in this region.

**Table 1:** Literature review.

Paper	Material and Methods	Main Results
*Acker et al., 2001* [24]	The paper analyzes the causes of the Balkan conflict linking food insecurity with political stability.	This research highlighted the role of agricultural education in post-conflict democratization.
*Papić-Brankov and Milovanović, 2015* [25]	Analysis of the Serbian food security system using Global Food Security Index Indicators.	The results showed the position of Serbia when food security is concerned and indicated two major weaknesses which influence the Serbian food system: low Gross Domestic Product per capita and corruption.
*Brankov and Lovre, 2017* [26]	The authors used the FAO package indicator to analyze the food security in the former Yugoslav republics.	The results showed that food security levels differ in analyzed countries, while Slovenia has the most favorable position when food security is concerned.
*Kovljenić and Raletić-Jotanović, 2020* [27]	Using multiple regression analysis, factors influencing food security in the former countries of Yugoslavia were analyzed.	The results showed that the general level of economic development, population growth, foreign trade, and agriculture investments significantly affect the level of food security. Additionally, results showed that Slovenia is the most nutritionally secure country, while the lowest level of food security has been observed in Bosnia and Herzegovina.
*Matkovski et al., 2020* [6]	Using the PROMETHEE method, Western Balkan and European Union countries are ranked according to food security. Also, factors affecting food security are estimated. FAO dimensions of food security are used.	The results show a significantly lower level of food security in the Western Balkan countries than in the EU. Also, results suggested that food security in the Western Balkans is not endangered, but it can become under crisis conditions.
*Božić and Nikolić, 2020* [28]	The food security system is analyzed using the Global Food Security Index for Serbia and neighboring countries: Bulgaria, Romania, Hungary, and Greece.	The results showed that Serbia lags behind the selected neighboring countries regarding food security.
*Brankov et al., 2021* [29]	The food self-sufficiency ratio is calculated for the South-East Europe countries. The influence of different factors affecting the self-sufficiency ratio is estimated.	The analyzed region showed a high level of food self-sufficiency, so it is ready to respond to crisis challenges. Also, this research highlighted regional cooperation, which should be strengthened.
*Đorđević et al., 2022* [30]	Theoretical presentation of food production in Serbia has been done since the arrival of Slavic tribes on the Balkan Peninsula up to modern times.	The results showed that although Serbia has been self-sufficient in food production throughout its history, there is space for improvement.
*Bogdanov et al., 2022* [31]	The report made a small attempt to evaluate the level of food self-sufficiency in the Western Balkan based on secondary data.	The results showed that all Western Balkan countries/territories, except Serbia, are net food importers.

Source. The authors’ composition.

Although a few studies analyze food security in the Western Balkan region (Table 1), they focus on FAO food security indicators—stability, availability, access, and utilization [6,26,28], not specifically on self-sufficiency. However, Bogdanov et al. [31] discussed food self-sufficiency ratio (SSR) and COVID-19 impacts on Western Balkan agri-food systems, but due to the lack of data, it was impossible to determine quantitively. Knowing the food self-sufficiency concept is not equal to the food security concept because food security is the state of having reliable access to a sufficient quantity of affordable and nutritious food regardless of whether the food is imported or domestically produced. Food self-sufficiency refers to the extent to which a country can satisfy its food needs from its domestic production [32], so this paper contributes to filling the gap in the literature about the region’s food self-sufficiency. As the region’s food security could be compromised due to restrictions in trade, own self-sufficiency must be paid more attention by the government as well as researchers.

## 2. Materials and Methods

Our sample is made up of five countries of the Western Balkan region (Albania, Bosnia and Herzegovina, North Macedonia, Montenegro, and Serbia (Data for the territory of Kosovo and Metohija is not reliable and often missing. Thus, this territory was excluded from the analysis)), over a 14-year period (2006–2019). The initial year of research is 2006 because it coincides with the gaining of independence of Montenegro. The end year (2019) is the most recent year available in the database. The selected time frame provides a good basis for exploring the region’s potential to satisfy its population’s food needs before the pandemic and the Ukrainian crisis and trade restrictions related to them (e.g., import of inputs necessary for production). It is essential to report the situation before the crisis in order to monitor the post-crisis situation in future research.

On the basis of the FAO calculation method [33], the food self-sufficiency ratio (SSR) is calculated by dividing the total domestic food output and the total supply in a certain country each year. Data on Food Balance from the FAOSTAT [34] is used in this analysis. SSR was calculated in the following way:

(1) $$\mathrm{SSR}=\frac{\mathrm{production}}{\mathrm{production}+\mathrm{import}-\mathrm{export}}\times 100\%$$

The countries with SSR < 100 produce less food than they consume. For example, if SSR is 25%, the country produces only 25% of the amount of food that it consumes. SSR = 100 means that the domestic food supply can satisfy domestic consumption; the country produces all amount of food that it consumes. On the other hand, the countries with SSR > 100 produce more food than they consume. For example, SSR = 140 means that 40% of the country’s production can be exported or placed in Commodity Reserves. SSR was calculated for food groups (cereals excluding total beer, starchy roots, total oil crops, fruits excluding wine, total vegetables, total sugar crops, total meat, total pulses, total eggs, milk excluding butter, total fish, and seafood) as well as for individual items: wheat, maize, barley, poultry, bovine, pig meat, mutton and goat, apples, grapes, tomatoes, onions, potatoes, beans, and sunflower oil, for each country, and the whole Western Balkan region. The same calculation method FAO use for the series of ‘cereal import dependency ratio.’ However, in the current crisis, which will potentially last for years, it is important to extend analyses to more commodities, not just cereals.

In the next step, we investigated the relationships between cereal production and population growth. After that, using data from the FAOSTAT database [34], we calculated fertilizers, pesticides, and irrigation efficiency in the region and the EU. Referring to Liu et al. (2015) [35], efficiency was calculated by dividing cereal production in the region by the annual application of fertilizers/pesticides/irrigated area. Instead of missing pesticide use data, we use data about the region’s pesticide import. The total area equipped for irrigation, global pesticide import, and consumption of total nutrient nitrogen, nutrient phosphate, and nutrient potash (N + P_2_O_5_ + K_2_O), was used as a proxy for fertilizers.

The achievement of food self-sufficiency in a country depends largely on input use, so the impact of different factors on the self-sufficiency of cereals was analyzed. The independent variables which are included in the model are arable land per capita, fertilizers efficiency, rural population, annual mean temperature change, annual precipitation increase, and GDP (Gross Domestic Products) per capita PPP (Purchasing Power Parity) change.

All variables were transformed into natural logarithms. The basic form of the estimated model is:

SSR_it_ = α + β1F_it_ + β2G_it_ + β3P_it_ + β4A_it_ + β5R_it_ + β6T_it_ + µ_i_ +λ_t_ + u_it_ (2)

where SSR_it_ represents SSR cereals in the Western Balkan country i in the period t; F_it_ represents fertilizers efficiency in the country i in the period t; G_it_ represents GDP per capita PPP in the country i in the period t; P_it_ represents precipitation in the country i in the period t; A_it_ represents arable land per capita in the country i in the period t; R_it_ represents rural population in the country i in the period t; T_it_ represents temperature change in the country i in the period t; µ_i_ and λ_t_ represent cross-section and period-specific effects (random or fixed), respectively; and u_it_ represents a random error of the model.

Knowing that the countries that lack natural resources (e.g., fertile land and water) are unable to achieve food self-sufficiency and depend on imports to provide food for their population [36], we use as a representative of natural resources variable—arable land (*A*). Variable temperature change (*T*) arose from several studies which showed that climate change could adversely affect the future availability of water and land, even in the case that their effects are milder than the effects of population change [37,38,39]. Interconnection between the rural population (*R*) and food self-sufficiency is most visible in the least developed countries that are all classified as net food importing countries [40]. However, 65.4% of its population resides in rural areas [41]. Production of cereals, especially corn, heavily depends on fertilizers and water [42]. That is why we include fertilizers efficiency (*F*) and annual precipitation increase (*P*) as variables. Usually, with economic growth, a country increases the ability to purchase food from abroad [43], so we included GDP per capita PPP (in constant 2017 international $) as a variable.

The selection of the appropriate panel model among the pooled Ordinary Least Square (OLS), Fixed-effect (FE), and Random-effect (RE) was based on the following tests: *Joint significance of differing group means, Breusch-Pagan*, and *Hausman* test statistic.

It is expected to find a positive relationship between the SSR of cereals and fertilizers efficiency (Table 2). It has been confirmed that fertilizer efficiency can be improved by applying proper management practices [44]. Enhanced efficiency of fertilizers can contribute to yield increase in various agricultural production. Water supply is another critical resource for cereal production, and reduced access to water can lead to productivity constraints [45]. Overall environmental benefits of increases in agricultural input use efficiency are larger than potential switches from conventional to alternative agricultural systems [46]. In order to produce food surplus, the countries have either a large amount of arable land per capita or a highly intensive production system [33]. Knowing that Western Balkans production is extensive in its essence, we can expect a positive influence of arable land per capita in the constructed model. Food insecurity is the highest among low-income and lowest in high-income countries [47], and rural people make up a high percentage of the hungry and malnourished [48], so special focus should be given to the policy of balanced rural development [49], which must focus on both the agricultural and non-agricultural sectors. They are often located on less favored agricultural land susceptible to low productivity [50]. Thus, it is expected to find a negative relationship between the SSR of cereals and the size of the rural population. Agriculture in the Western Balkans is considered critically affected by climate change [51]. That is, we included annual temperature change as a variable. Temperature, via weather extremes—droughts and heat wave—strongly affects agricultural production [52], so it is expected that temperature changes negatively affects cereals self-sufficiency. Considering the small participation of the irrigated area in the total agricultural area [34,53,54] it is expected to find that precipitation increase positively affects cereal self-sufficiency. Finally, it is expected to find a negative relationship between GDP per capita and SSR because, usually, with the growth of the population’s purchasing power, countries import more [43].

As shown in Table 2, the main sources of data are FAOSTAT [34] and World Bank [55].

## 3. Results

### 3.1. Food Self-Sufficiency

**Figure 1:**
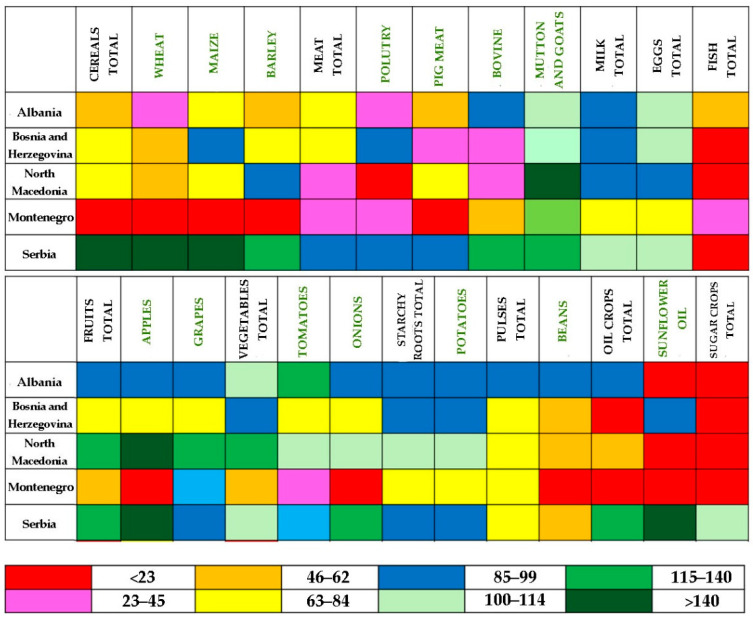
Heatmap, Self-sufficiency ratio based on FAOSTAT (average five years). Source. The authors’ calculation.

Calculation of the 5-year average food SSR showed that out of five Western Balkans countries, only one country (Serbia) had an overall SSR above 100% for most items (Figure 1). The other analyzed countries showed SSR below 100% for many analyzed products.

A list of the Western Balkans’ countries ranked by the self-sufficiency level is as follows: Serbia is ranked 1st, North Macedonia 2nd, Albania 3rd, Bosnia and Herzegovina 4th, and Montenegro 5th. **Serbia** is self-sufficient in the following items: all cereals, including corn, wheat, and barley; some meat—bovine and mutton and goat; milk; eggs; fruits total and some kind of fruits (apples); vegetables total and some individual vegetables item (onions); sugar crops; and sunflower oil. **North Macedonia,** unlike Serbia, is highly dependent on cereals and meat imports. It imports about 30% of its cereals needs, almost half of its wheat needs, and more than 70% of its meat. The most critical situation is with poultry—an SSR is just about 7%. However, the country produces an abundance of fruits and vegetables and enough starchy roots to satisfy the demand of its population. Just like North Macedonia, **Albania** is dependent on cereals and meat imports. It imports about 60% of the wheat, 20% of corn, 40% of barley, 40% of poultry, and 40% of pig meat needs. Unlike North Macedonia and Serbia, it is not self-sufficient in fruits, even though it is close to reaching self-sufficiency in apples and grapes. The vegetable sector has better performance, and in addition to Serbia and North Macedonia, Albania has an overall SSR for vegetables (especially tomatoes) above 100%. The situation in **Bosnia and Herzegovina** is hard to explain—Bosnia and Herzegovina consists of two Entities: the Republic of Srpska and the Federation of Bosnia and Herzegovina. For example, the Federation of Bosnia and Herzegovina is dependent on wheat imports, while the Republic of Srpska produces enough wheat to meet the demand. The whole country is self-sufficient only in mutton and goat meat and eggs. In other words, this country relies on imports for almost all foods. It imports more than half of wheat needs, above 10% of corn and 20% of barley, somewhat below 40% of meat total, more than 70% of pig meat and above 60% of bovine, nearly 90% of sugar and oil crops, almost 30% of fruits, 10% of vegetables, more than 30% of tomatoes, 25% of pulses, 10% of starchy roots, etc. The 5th ranked **Montenegro** is the most dependent country on food imports. It produces only sufficient quantities of mutton and goat meat to meet the demand. The country produces only 5% of cereals demand, 25% of meat, about 70% of milk and eggs, somewhat above 55% of fruits and vegetables, 72% of potatoes, etc. Although Montenegro has a significant coastline, it produces only 26% of its fish needs.

**Figure 2:**
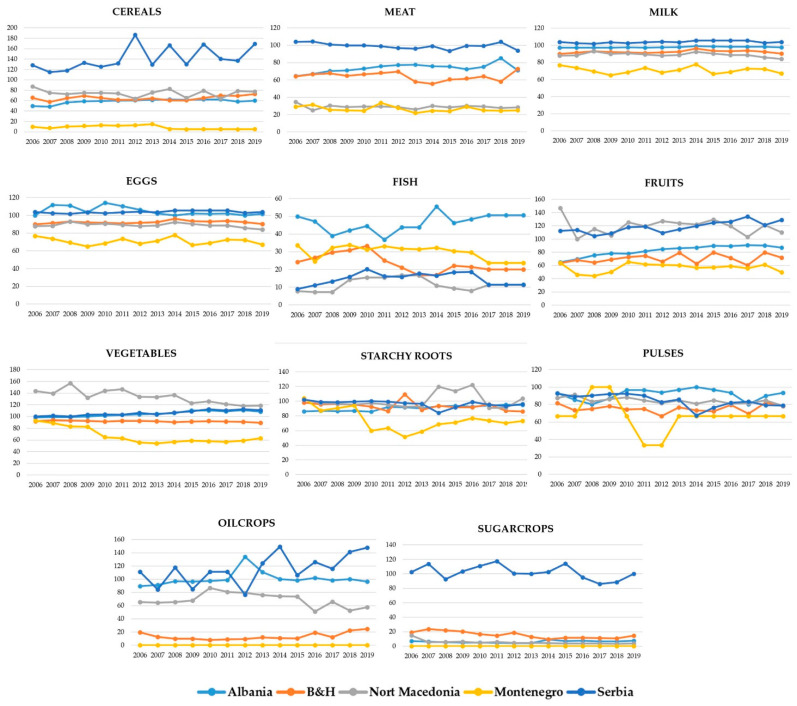
Self-sufficiency ratio based on FAOSTAT. Source. The authors’ calculation.

Tendencies on SSR are shown in Figure 2. As can be seen from Figure 2, the general tendencies of SSRs in the observed period (2006–2019) are as follows: (1) Albania increased SSR levels of cereals, meat, eggs, vegetables, fruits, oil crops, and starchy roots for about 10%, 5%, 1%, 9%, 22%, 6%, and 8%, respectively, while no significant changes were observed in SSR of pulses, fish, milk, and sugar; (2) Bosnia and Herzegovina, increased SSR level of cereals, eggs, fruits, and oil crops by 12%, 19%, 8%, and 5%, respectively. The level of vegetables, starchy roots, pulses, fish, and sugar decreased by about 2%, 12%, 3%, 4%, and 7%, respectively. SSR level of meat and milk remain more or less the same in this country; (3) In North Macedonia, the SSR level of fish increased by about 3% and starchy roots by about 2%. The level of SSR of cereals, meat, eggs, vegetables, pulses, fruits, sugar crops, and oil crops decreased by about 10%, 6%, 22%, 25%, 9%, 37%, 11%, and 5%, respectively. SSR of milk remains more or less the same; (4) Montenegro increased only the level of SSR of eggs by about 5%. The level of SSR of pulses, sugar crops, and oil crops remained the same. The decrease is observed in the level of SSR of cereals and meat (about 4%), milk and fish (about 10%), vegetables and starchy (about 30%), and fruits (about 14%); (5) Serbia increased level of SSR of cereals, meat, fish, vegetables, fruits, and oil crops by about 41%, 10%, 2%, 10%, 14%, and 36%, respectively. In the observed period, the country decreased the SSR of starchy roots and pulses by 6% and 14%, respectively, while there were no significant changes in the SSR of eggs, milk, and sugar crops.

**Figure 3:**
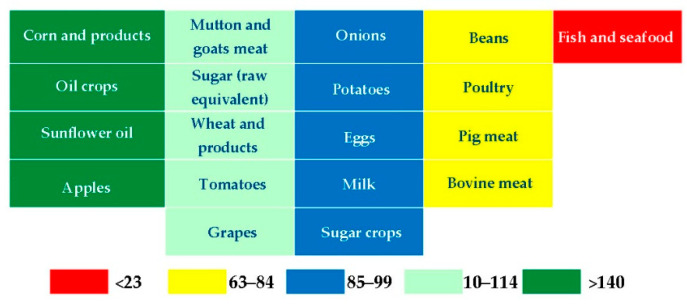
Heatmap, Self-sufficiency in the Western Balkan region as a whole. Source. The authors’ calculation.

Thanks to Serbia, the region as a whole is well supplied with many agricultural products (Figure 3). The region produces corn, apples, and sunflower oil for its needs and export; it is self-sufficient in wheat, tomatoes, grape, sugar, and mutton and goat meat. Apart from the fish, the region is 20–25% dependent on pig, bovine, and poultry meat imports. Generally, the level of food security in Western Balkan countries is not endangered, but it is lower than in EU countries, and it could be endangered in the crisis conditions [6]. Additionally, some research showed that the Western Balkan region is quite ready to respond to the challenges in the crisis conditions, but regional cooperation needs to be strengthened [29], and one of the steps is the initiative Open Balkan which can intensify the trade, free movement and economic development [56]. The CEFTA Agreement significantly improved foreign trade of agri-food products in the Western Balkan countries [57], so the initiative Open Balkan is the next step in this process of better regional cooperation.

The region is characterized by long-term stagnation in livestock production. According to Statistical Office of the Republic of Serbia (SORS) data, in Serbia, livestock has been halved in the last 30 years. In the period 2006–2019, the number of pigs decreased from 3999 to 2868 thousand, and the number of cattle from 1106 to 860 thousand [58]. In enhancing livestock production, appropriate state support is crucial [59]. The same can apply to all other countries in the region.

### 3.2. Cereals Production and Demand

The current global population is 7.97 billion, and it is expected to be 9.7 billion in 2050 [60]. Rates of population growth vary significantly across countries and regions, whereas the populations of the 46 least developed countries (LDCs) are expected to experience double growth in population between 2022 and 2050 [60]. In order to meet human needs, global cereal production has to be increased by an additional quantity of 0.3 billion tons in the next ten years (from 2.8 billion tons to 3.1 billion tons) [61]. This would put additional pressure on resources [60], so apart from increasing the amount of food produced, it is also vital to provide efficiency in production and equity in distribution [62].

**Figure 4:**
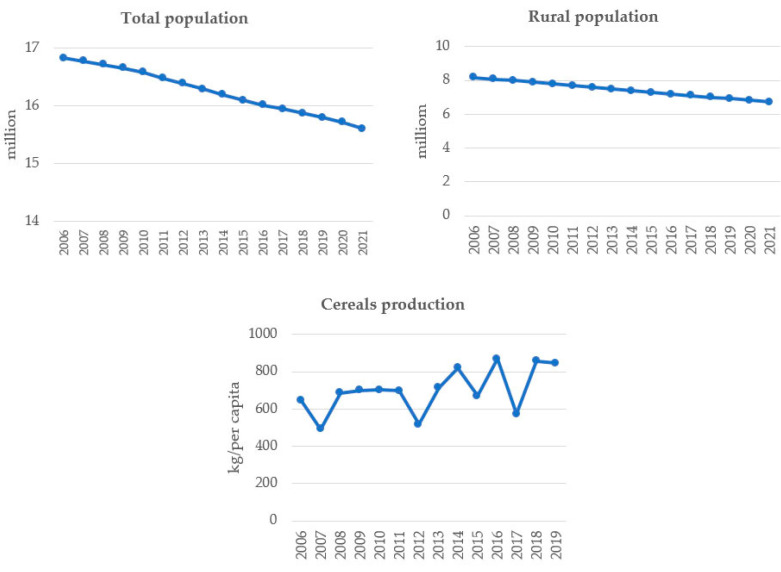
Evolution of total and agriculture population and cereal production in the Western Balkans. Source. The authors’ presentation on the basis of data from the World Bank [55].

In the Western Balkans, during the observed period, the annual population decreased by an average annual growth rate (AAGR) of −0.005, while the rural population decreased by an AAGR of −0.013. In the same period, cereal production increases with 0.049 AAGR (Figure 4). Cereal production in the Western Balkans increased from 646.5 to 845.8 kg/pc from 2006–2019, which is significantly above the average annual cereal human consumption of 175 kg globally [63], and above the cereals harvested per each EU inhabitant (583 kg in 2019). The analysis showed negative population growth and positive cereals production growth in the Western Balkans, suggesting that the region is in a better position than many others in the world.

**Figure 5:**
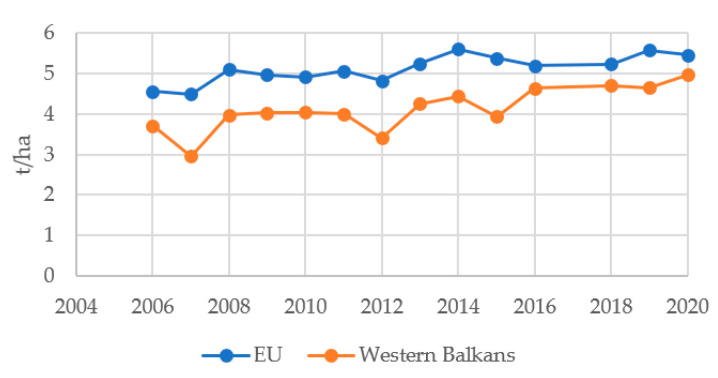
Cereal yield in the Western Balkans and the EU. Source. The authors’ presentation on the basis of data from the World Bank [55].

Western Balkans use about two times less fertilizer (N, P, K) for its agricultural production than the EU (the EU: 157.2 kg/per ha of arable land in 2018; Western Balkans: 73.9 kg); imports almost four times less pesticides (14.5 kg/ha vs. 3.7 kg/ha in 2019); and has a lower share of land area equipped for irrigation (18.8% vs. 11.3%), but have just less than 10% lower cereals yield than the EU [34] (Figure 5). AAGR for the cereals yield in the EU was 0.015, and twice as big in the WB −0.033. More importantly, the difference in yields between the two regions is increasingly decreasing in favor of the Western Balkans.

### 3.3. Fertilizers, Pesticides, and Irrigation Efficiency

**Figure 6:**
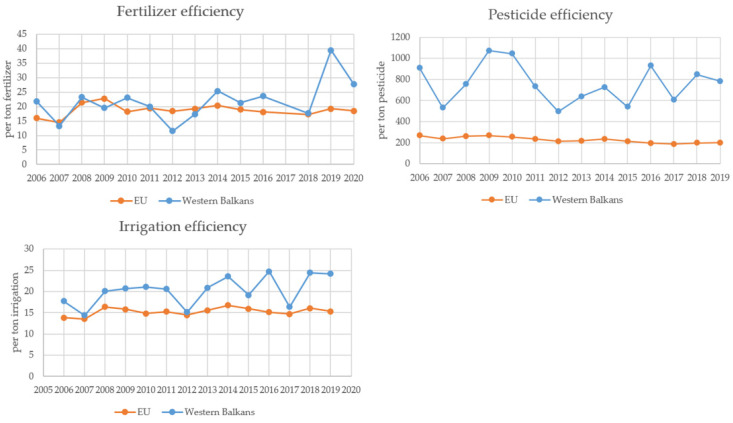
Inputs efficiency in the Western Balkans and the EU. Source. The authors’ calculation.

After numerous variations, since 2014, **fertilizers efficiency** has been continuously higher in the Western Balkans than in the EU. After 2009, fertilizers efficiency in the EU decreased by 19% for each ton of fertilizers produced, from 22.8 tons of cereal produced (per ton fertilizer) in 2019 to 18.5 tons of cereal (per ton fertilizer) in 2019. In the same period, fertilizers efficiency in the Western Balkans increased by 42.3%, from 19.5 to 27.8 tons of cereals produced per ton of fertilizer (Figure 6). A decrease in fertilizers efficiency is in line with the global trend and can be caused by decreased plants’ sensitivity to fertilizers or changes in modern farming practices [35].

**Pesticide efficiency** is almost four times lower in the EU than in the Western Balkans. Pesticide efficiency decreased by 25% in the EU, in the period 2006–2019, per ton of pesticide import while remaining relatively stable in the Western Balkans. Decreases in pesticide efficiency in the EU partially resulted from evolutionary interactions among crops, insects, weeds, and pathogens [35] and partially due to its overuse. The EU is the world’s largest pesticide consumer [64]. Contrary, countries in the region are not among the largest users of pesticides due to the extensiveness of their agriculture [65].

The **irrigation efficiency** in the EU in the period 2006–2019 increased by 10%, while the Western Balkan region made a significant improvement—a 36% increase. However, recognizing that climate change will increasingly affect agriculture that relies on rainfall, increases in irrigation efficiency need to continue using water resource management amid climate change [66].

### 3.4. Econometric Estimation of Input Use

Based on panel diagnostic tests (Table 3), we choose RE in favor of FE and OLS models. Significant differences between conventional standard errors and robust standard errors show that the model suffers from heteroskedasticity. With respect to serial correlation (autocorrelation), the Durbin–Watson statistic value shows that there is a first-order autocorrelation. So, to deal with the problems of heteroskedasticity and serial correlation, we performed the Weighted Least Squares (WLS) method as the most appropriate to have efficient estimators [29].

Based on the results of the panel analysis Model I (Table 4), arable land per capita, rural population, and fertilizers efficiency had a significant and positive effect on cereals self-sufficiency, while temperature change had a negative and significant effect (Table A1). Precipitation increase had a positive but non-significant effect. Thus, in Model II, we drop this variable (Table A2). Instead, we add GDP per capita PPP. Here again, we got the same sign of the previously observed variables. As expected, we got a negative and significant effect of GDP increase on SSR.

## 4. Discussion

Western Balkan countries varied considerably by the level of food self-sufficiency. However, the region as a whole can fulfill its population’s feed demand. The greatest merit for ensuring food security belongs to Serbia, which is the only net exporter of agricultural goods in the region [67,68]. Although there is a need to increase the productivity of the Serbian agri-food sector by increasing the participation of high-value food in the total export [69], Serbia so far successfully plays the role of the region’s key supplier. Despite all past and current problems, the region is ready to respond to the challenges posed by the crisis. Of course, a prerequisite is to improve cooperation and reconciliation. This finding is in line with previous research [29].

The results of this research also bring certain warnings, especially for the livestock sector. The region cannot satisfy the meat needs of its population—it is 20–25% dependent on pig, bovine, and poultry imports. For example, in Serbia, the livestock has been halved in the last 30 years. The number of cattle, pigs, sheep, and poultry declined from 1559, 4238, 2120, and 23,405 to 860, 2868, 1695 and 15,348, respectively, in the period 1990–2021 [70]. A similar trend is observed in other regions country, especially in North Macedonia, where dairy farms operate under high uncertainties in an unregulated institutional environment [71], or in Bosnia and Herzegovina—there were 970,142 cattle before the war, and after the war, the number of cows decreased to 218,406 [72]. The problems that threaten self-sufficiency in the meat sector are systemic and have distant roots in the 1990s. They are linked to the post-war institutional and economic reforms that caused an initial decline in agricultural productivity caused by the privatization of production capacity, land reforms, farm restructuring, budget constraints, lack of incentives, and disorganized food supply chains.

Contrary to the global and the EU trend of decreasing fertilizers and pesticide efficiency [35], the Western Balkans showed a satisfactory level of input efficiency. Most probably, this is due to the extensive nature of its agriculture. However, this finding supports the sustainable production of the region and is very important because food production in the coming decades will focus on enhancing input efficiency [35].

Five indicators are very important for the cereals’ self-sufficiency in the region: arable land, rural population, fertilizers efficiency, temperature change, and GDP per capita PPP increase. The positive effect of arable land and fertilizer efficiency on SSR obtained in our work is in line with previous research [33,46]. Contrary, the rural population had the opposite effect of what had been predicted, suggesting that the region’s agriculture relies on natural resources and workforce rather than new technologies and investments. This is in line with the research of Lovre (2016) [73] about a slowdown in the growth of agricultural investment in the Western Balkans. Namely, this research showed a deceleration in the total factor productivity growth and an acceleration in input growth, which influenced the slow growth of the real output of agriculture. In addition, the negative effect of temperature change and GDP increase is also in line with previous research [36,39,43].

The information that fertilizers efficiency highly contributes to achieving self-sufficiency is very important in the circumstances of the current crisis. Western Balkan region depends on fertilizers import. According to ICT statistics [74], in 2020, Serbia imported 365,127 tons of mineral or chemical fertilizers containing two or three fertilizing elements (code 3105), of which 85% came from the Russian Federation. Albania imported 44,660 tons of fertilizers (code 3105) mainly from Greece (29.9%) and Tunisia (27.5%); Bosnia and Herzegovina 42,333 tons (37% from Croatia and 24% from Russia); North Macedonia 21,465 tons of which 37.2% from Greece and 33.5% from Serbia; while Montenegro imported 5029 tons of fertilizers (32.6% from Serbia, 21.7% from Croatia). So that agricultural production does not suffer, domestic capacities in producing fertilizers must be strengthened, and proper farming practices and management techniques to increase the sensitivity of plants to fertilizers should be applied.

Regarding the specific impact of each one of the climatic variables, results indicate that rainfall has a positive but non-significant effect, while temperature has a negative significant impact. In fact, temperature changes are already critical, which is in line with research [51]. However, this is an emerging problem that will become increasingly apparent in the coming years if we take into account the dependence of production on rainfall and the small percentage of irrigated areas. Out of 553,000 hectares of the region’s land area equipped for irrigation, 64.5% belongs to Albania, 0.5% to Bosnia and Herzegovina, 23.1% to North Macedonia, 0.5% to Montenegro, and 11.4% to Serbia. On the other side, in the whole region’s cereals production (about 13.4 million tons), Albania participates with about 5%, Bosnia and Herzegovina 12.5%, North Macedonia 4.2%, Montenegro 0.05%, and Serbia with 78.3%. In other words, the two countries (Albania and North Macedonia) most equipped for irrigation (87.6% of the total region’s irrigated area) account for just 9.2% of the total region’s cereals production. In contrast, the region’s leading cereals supplier (Serbia) participates in the region’s irrigated area with just 11.4% [34]. Irrigation systems in Albania and North Macedonia are used for vegetable production, while cereals occupy an inconsiderable place. In other words, cereals production in the Western Balkans is extensive and exposed to weather conditions.

Food self-sufficiency should not be a goal at any price and cannot be equated with food security. For example, despite the high level of food self-sufficiency in Serbia (the best-ranked country in our sample), there is a downward trend in economic access to food. Even more, the country has the second highest population living hunger in Europe (5.7% undernourishment rate) [75]. Clapp (2017) [76] pointed out four key risks associated with food autarky: production variability, market distortions and consequently high food prices, the reduced income of farmers producing for export, and environmental challenges. On the other side, the author highlighted that some groups of countries: poor countries, those with volatile export earnings or with an abundance of natural resources and with a large population, the countries whose food system is controlled by a small group of suppliers, as well as countries at risk of war or political tensions could reap numerous benefits if they reduce their dependence on food imports. Taking into account the specificities of each national food system and the right of the country to define its food policy, one can take a balanced position between two contradictory approaches—food autarky and fully open borders.

Out of all Western Balkan countries, the Ukrainian crisis hit Albania hardest since it imports significant quantities of its total wheat from Russia. For example, in 2020, about 62% of wheat import was realized from Russia [77]. However, in the Open Balkan initiative, Serbia agreed to export cereals and products to Albania as a rescue. As a result, awareness about regional cooperation’s role in insulating the region from international supply disruptions is getting stronger. Furthermore, the significant food price increase and dependence on importing certain food and agrochemicals stimulate awareness-raising on food self-sufficiency in the region.

The findings of this study can be useful for policymakers to define appropriate responses to boost food and nutrition security, reduce risks, and strengthen food systems. Most of all, in order to increase the region’s food security, the countries in the Western Balkan have to reinforce intra-regional trade cooperation. For example, for effective emergency management, an establishment of the Crisis committee (by ministries of agriculture), improvement in information sharing and data collection, development of the system of a rapid assessment of the food stocks, etc., are highly recommended.

## 5. Conclusions

The COVID-19 pandemic started in 2020 and significantly influenced food security in the world. Additionally, the conflict in Ukraine has deepened this problem, as it has blocked ports, the prices of freight and fertilizer costs increased, the risk to the global economy is higher, the number of refugees increased, and many countries emptied their food reserves. In that context, the main idea of this paper was to estimate the Western Balkans’ food security and answer the question of whether food shortage is coming to this region.

This research showed that this region, as a whole, is well supplied with the majority of food products. This is thanks to Serbia, the only country with an overall SSR above 100% for the most analyzed items. So, the level of self-sufficiency varies across the Western Balkan countries, where Serbia is in the first position while Montenegro is the worst. Because of that, regional cooperation is crucial, and the Open Balkan initiative is one step forward to better cooperation, signed by Serbia, Albania, and North Macedonia. Although results showed that the region is well supplied with food products, it could be endangered in the crisis conditions. For example, bans on the export of certain types of basic foodstuffs from Serbia at the beginning of the Ukrainian crisis significantly disrupted the situation on the market. Therefore, in crisis conditions, certain situations can threaten the efficient supply of food to the market at any moment. This can be very pronounced in livestock production that has retrograde tendencies.

Additionally, the results of this research showed a satisfactory level of input efficiency, most probably due to the extensive nature of agriculture in the Western Balkan region. This is very important for the continuation of sustainable food production, which will surely focus more and more on the efficiency of inputs in the coming period.

Factors significantly influencing cereals’ self-sufficiency in the Western Balkans are arable land, rural population, fertilizers efficiency, temperature change and GDP increase. In the context of crisis conditions, it is valuable information that fertilizers efficiency significantly contributes to cereals’ self-sufficiency level, as the Western Balkans depend on the import of fertilizers. So, in this situation, all countries of this region need to strengthen domestic capacities in producing fertilizers to enable continuous food production.

This research has limitations. Some factors, such as the role of trade policies, infrastructure, food prices, and agriculture’s role in the overall economy that can affect food supplies and food self-sufficiency, have not been included in the statistical analysis. Thus, future research can be directed toward a more detailed analysis of factors influencing regional self-sufficiency. Also, further study is required to quantify the effect of Open Balkan (and/or similar initiatives) to discuss in detail the common regional market, pros, and cons. In order to achieve the highest possible self-sufficiency level, the investigation of the current diets, their modification, and the reduction of food loss and waste should be imperative. On the whole, future research can be directed toward a more detailed analysis of factors influencing self-sufficiency in the region.

## Figures and Tables

**Table 2:** Explanatory variables.

Variable	Description	Source	Expected Relationship
*F*	Fertilizers efficiency	FAOSTAT	Positive
*G*	GDP PPP	World Bank	Negative
*P*	Precipitation	World Bank	Positive
*A*	Arable land	World Bank	Positive
*R*	Rural population	World Bank	Negative
*T*	Temperature change	World Bank	Negative

Source. The authors’ composition.

**Table 3:** Panel diagnostic test.

Dependent Variable	Joint Test on Named Regressors	Breusch–Pagan Test Statistic	Hausman Test Statistic
SSR_cereals	Chi-square (6) = 91.3096 *p*-value = 0.0000	Chi-square (1) = 12.2268 *p*-value = 0.216493	Chi-square (4) = 3.36118 *p*-value = 0.499297

Source. The authors’ calculation.

**Table 4:** Model estimation of SSR for the Western Balkan countries.

	Model I	Model II
Const	0.00770808	0.0109142
Fertilizers efficiency	0.380233 ***	0.337896 ***
GDP PPP		−0.0590066 **
Precipitation	0.0406102	
Arable land	0.114664 ***	0.119326 ***
Rural population	0.655492 ***	0.691550 ***
Temperature change	−0.0588534 **	−0.0517610 *

Note: *, **, and *** level of significance are 10%, 5%, and 1%, respectively. Source. The authors’ calculation.

## Data Availability

All databases are available for use and the public.

## Appendix A

**Table A1:** Estimation of Model I (WLS).

	Coefficient	Std. Error	t-Ratio	*p*-Value	
const	0.00770808	0.0340386	0.2265	0.8223	
Fertilizers efficiency	0.380233	0.0613847	6.194	<0.0001	***
Precipitation	0.0406102	0.0336786	1.206	0.2367	
Arable land	0.114664	0.0361433	3.172	0.0033	***
Rural population	0.655492	0.0517909	12.66	<0.0001	***
Temperature change	−0.0588534	0.0261677	−2.249	0.0315	**
Statistics based on the weighted data:					
Sum squared resid		35.31887	S.E. of regression		1.050578
R-squared		0.979263	Adjusted R-squared		0.976022
F(5, 32)		302.2225	*p*-value(F)		6.12 × 10^−26^
Log-likelihood		−52.52946	Akaike criterion		117.0589
Schwarz criterion		126.8844	Hannan-Quinn		120.5548
Statistics based on the original data:					
Mean dependent var		−0.107951	S.D. dependent var		1.120360
Sum squared resid		2.103051	S.E. of regression		0.256360
Test for normality of residual— Null hypothesis: error is normally distributed Test statistic: Chi-square (2) = 4.24099 with *p*-value = 0.119972					
Pesaran CD test for cross-sectional dependence— Null hypothesis: No cross-sectional dependence Asymptotic test statistic: z = 0.746223 with *p*-value = 0.455533					

*Note: **, and *** level of significance 5%, and 1%, respectively*. Source. The authors’ calculation.

**Table A2:** Estimation of Model II (WLS).

	Coefficient	Std. Error	t-Ratio	*p*-Value	
const	0.0109142	0.0340949	0.3201	0.7510	
Fertilizers efficiency	0.337896	0.0601721	5.615	<0.0001	***
GDP PPP	−0.0590066	0.0289188	−2.040	0.0496	**
Arable land	0.119326	0.0341963	3.489	0.0014	***
Rural population	0.691550	0.0495851	13.95	<0.0001	***
Temperature change	−0.0517610	0.0260480	−1.987	0.0555	*
Statistics based on the weighted data:					
Sum squared resid		33.68589	S.E. of regression		1.026004
R-squared		0.981108	Adjusted R-squared		0.978156
F(5, 32)		332.3715	*p*-value(F)		1.38 × 10^−26^
Log-likelihood		−51.63003	Akaike criterion		115.2601
Schwarz criterion		125.0856	Hannan-Quinn		118.7559
Statistics based on the original data:					
Mean dependent var		−0.107951	S.D. dependent var		1.120360
Sum squared resid		2.068543	S.E. of regression		0.254248
Test for normality of residual— Null hypothesis: error is normally distributed Test statistic: Chi-square (2) = 1.62587 with *p*-value = 0.443554					
Pesaran CD test for cross-sectional dependence— Null hypothesis: No cross-sectional dependence Asymptotic test statistic: z = −1.04505 with *p*-value = 0.295999					

*Note: *, ** and *** level of significance are 10%, 5% and 1%, respectively*. Source. The authors’ calculation.
